# Sugar Phosphorylation Controls Carbon Source Utilization and Virulence of *Candida albicans*

**DOI:** 10.3389/fmicb.2020.01274

**Published:** 2020-06-16

**Authors:** Stefanie Wijnants, Michael Riedelberger, Philipp Penninger, Karl Kuchler, Patrick Van Dijck

**Affiliations:** ^1^Laboratory of Molecular Cell Biology, Department of Biology, Institute of Botany and Microbiology, KU Leuven, Leuven, Belgium; ^2^VIB-KU Leuven Center for Microbiology, Leuven, Belgium; ^3^Max Perutz Labs Vienna, Center for Medical Biochemistry, Medical University of Vienna, Vienna, Austria

**Keywords:** *Candida albicans*, glycolysis, hexokinase, glucokinase, virulence, central metabolism

## Abstract

*Candida albicans* is an opportunistic human fungal pathogen that relies upon different virulence traits, including morphogenesis, invasion, biofilm formation, and nutrient acquisition from host sources as well as metabolic adaptations during host invasion. In this study, we show how sugar kinases at the start of glycolysis modulate virulence of *C. albicans*. Sequence comparison with *Saccharomyces cerevisiae* identified four enzymes (Hxk1, Hxk2, Glk1, and Glk4) in *C. albicans* with putative roles in sugar phosphorylation. Hxk2, Glk1, and Glk4 demonstrate a critical role in glucose metabolism, while Hxk2 is the only kinase important for fructose metabolism. Additionally, we show that Hxk1 controls *HXK2*, *GLK1*, and *GLK4* expression in the presence of fermentable as well as non-fermentable carbon sources, thereby indirectly controlling glycolysis. Moreover, these sugar kinases are important during virulence. Disabling the glycolytic pathway reduces adhesion capacity, while deletion of *HXK1* decreases biofilm formation. Finally, we demonstrate that *hxk2Δ*/*Δ glk1Δ/Δ glk4Δ/Δ* and *hxk1Δ/Δ hxk2Δ/Δ glk1Δ/Δ glk4Δ/Δ* have attenuated virulence upon systemic infections in mice. These results indicate a regulatory role for Hxk1 during sugar phosphorylation. Furthermore, these kinases are essential during growth on glucose or fructose, and *C. albicans* relies on a functional glycolytic pathway for maximal virulence.

## Introduction

*Candida albicans* is an opportunistic fungal pathogen. It is the predominant causal agent of invasive candidiasis and the fourth leading cause of hospital-acquired infections (Perlroth et al., 2007). This pathogen can survive and grow in distinct host environments and is present as a commensal colonizer on epithelial barriers of the human body, including oral mucosa, gastrointestinal flora, and genitalia (Ganguly and Mitchell, 2011; Fox et al., 2013; Richardson et al., 2019).

*Candida albicans* has different virulence factors to colonize the human body, including morphogenesis, invasion, white-opaque switching, biofilm formation, and several fitness traits (Liu, 2002; Hofs et al., 2016). In patients with a weakened immune system, *C. albicans* can disseminate into the human body, mainly by reversibly switching morphology between the yeast and hyphal form in order to penetrate tissues. The capacity to change morphology and break barriers is critical for dissemination through the body, as strains unable to switch have a strongly reduced virulence (Whiteway and Bachewich, 2007). Another important virulence factor is the formation of biofilms, which is a complex community of fungal cells attached to biotic or abiotic surfaces and embedded in a matrix (Řičicová et al., 2010; Finkel and Mitchell, 2011). Adhesion of *C. albicans* cells to a surface is an important initial step during biofilm formation, and different proteins and regulators such as Efg1 and Eap1 play essential roles in this adhesion process (Finkel and Mitchell, 2011). Importantly, fungal cells embedded in a mature biofilm show a higher tolerance to the antifungal drug fluconazole. This is due to the higher expression of multidrug efflux pumps and by the fact that the drug can become trapped in the extracellular matrix (Mateus et al., 2004; Taff et al., 2012).

*Candida albicans* virulence traits are under tight control of different signaling pathways, including the external pH-regulated Rim101 pathway, the protein kinase A (PKA) pathway, and the mitogen-activated protein kinase (MAPK) pathway. These last two are important for growth, morphogenesis, and adhesion (Davis, 2003; Biswas et al., 2007; Han et al., 2011). Both the PKA and the MAPK pathways are activated and stimulated by different external stimuli, including glucose, amino acids, Co_2_levels, and temperature as well as host oxidative defense molecules (Biswas et al., 2007; Broach, 2012; Desai et al., 2015). Glucose availability affects the activation of the PKA pathway, but the exact mechanism how glucose activates PKA is not completely understood. It is known that Cdc25 and Ras1 play an important role during this process, since strains lacking one of these two proteins fail to accumulate cAMP upon glucose availability (Maidan et al., 2005).

The central carbon metabolism also plays pivotal roles in *C. albicans* pathogenicity, since a clear link between carbon control and pathogenicity is observed (Magalhães et al., 2017; Thammahong et al., 2017; Van Ende et al., 2019; Lagree et al., 2020). For example, it is shown that the amount of trehalose in the cells has an effect on filamentation (Serneels et al., 2012). Also, enzymes involved in the glucose repression mechanism have an effect on virulence, since two transcription factors, Mig1 and Mig2, involved in this pathway have a dual role. They inhibit the expression of genes necessary for the utilization of alternative carbon sources, and they are responsible for the expression of genes involved in virulence traits, like filamentation and biofilm formation (Lagree et al., 2020). A third example of a link between carbon source and pathogenicity is the fact that glycolysis is upregulated during infections and thereby strongly contributes to the virulence of this pathogenic fungus (Barelle et al., 2006). Additionally, the expression of glycolytic genes is regulated in response to different environmental conditions, for example, their expression is upregulated during oxygen limitation (Setiadi et al., 2006; Askew et al., 2009). Also, when circumstances favor the commensal-to-pathogen switch, glycolytic genes are upregulated and *C. albicans* becomes virulent. Indeed, the deletion of *GAL4* and *TYE7*, which bind to the promoters of glycolytic genes to activate their expression, causes a growth defect and attenuated virulence on fermentable carbon sources (Askew et al., 2009).

At least 20 different hexose transporters, Hgt1-Hgt20, are found in *C. albicans*. They show high homologies when compared to the human GLUT transporters and the *Saccharomyces cerevisiae* Hxt transporters (Fan et al., 2002). Several transporters are under control of the glucose sensor Hgt4, which senses glucose, fructose, and mannose, and is only expressed under limiting glucose concentrations (Brown et al., 2006). Hgt4 may be the ortholog of the yeast Rgt2 and Snf3 glucose sensors. In baker’s yeast, glucose is rapidly phosphorylated into glucose-6-phosphate upon cellular uptake and metabolized into pyruvate during glycolysis. During the course of our work, a paper was published where they analyzed possible enzymes involved in this glucose phosphorylation step. They identified three kinases, Hxk2, Glk1, and Glk4, which were responsible for the phosphorylation of glucose (Laurian et al., 2019). These results are similar to our data, although, based on phylogenetic analysis, we found four ortholog enzymes compared to sugar kinases in *S. cerevisiae*, Hxk1, Hxk2, Glk1, and Glk4. Therefore, we also included Hxk1 in our study since this enzyme can also have an effect on sugar phosphorylation in *C. albicans* based on its structure. *HXK1* is located in the NAG regulon, together with *DAC1* and *NAG1*, and the three encoded enzymes are responsible for the conversion of N-acetylglucosamine (GlcNAc) into fructose-6-phosphate (Kumar et al., 2000). Strains lacking *HXK1* fail to grow on GlcNAc, but have no growth problem on glucose as a carbon source. The *hxk1Δ/Δ* strain is hyperfilamentous and shows upregulated opaque genes, implying that virulence and morphogenesis could be affected (Naseem et al., 2011; Rao et al., 2014; Cao et al., 2016). Hxk2 is upregulated by filamentation stimuli and in fluconazole-treated cells, although its expression is downregulated by hydrogen peroxide (Ebanks et al., 2006; Liang et al., 2011; Monteoliva et al., 2011). Hxk2 is under control of the sensor/receptor repressor (SRR) pathway and thus regulated by Hgt4 and Rgt1 (Brown et al., 2006; Van Ende et al., 2019). Of note, the function of the putative glucokinases Glk1 and Glk4 is unknown, although both are upregulated in primary macrophages, and Glk1 responds to hypoxic conditions (Marcil et al., 2008; Synnott et al., 2010).

Here, we report that Hxk2 and Glk1/4 are responsible for the conversion of glucose into glucose-6-phosphate, while only Hxk2 converts fructose into fructose-6-phosphate. Furthermore, we show that Hxk1 regulates the expression of *HXK2* and *GLK1/4*, and we demonstrate that Hxk2 affects the glucose transport of *C. albicans* consistent with a recent report (Laurian et al., 2019). Finally, deletion of kinases or disruption of the glycolytic pathway attenuates the virulence of *C. albicans*. Impaired glycolysis affects *C. albicans* adhesion, while deletion of *HXK1* reduces biofilm formation. Finally, we demonstrate that the *hxk2Δ/Δ glk1Δ/Δ glk4Δ/Δ* and the *hxk1Δ/Δ hxk2Δ/Δ glk1Δ/Δ glk4Δ/Δ* mutants are avirulent during a systemic infection in mice.

## Materials and Methods

### Strains

The strains used in this study are presented in Table 1.

**TABLE 1:** Strains used in this study.

Strains	Relevant Genotype	Obtained from
SC5314		Gillum et al., 1984
*hxk1Δ/Δ*	*hxk1Δ*::FRT*/hxk1Δ*::FRT	This work
*hxk2Δ/Δ*	*hxk2Δ*::FRT*/hxk2Δ*::FRT	This work
*hxk1Δ/Δ hxk2Δ/Δ*	*hxk1Δ /hxk1Δ hxk2Δ*::FRT*/hxk2Δ*::FRT	This work
*glk1Δ/Δ glk4Δ/Δ*	*glk1Δ/glk1Δ glk4Δ/glk4Δ*	This work
*hxk1Δ/Δ glk1Δ/Δ glk4Δ/Δ*	*hxk1Δ*::FRT*/hxk1Δ*::FRT *glk1Δ/glk1Δ glk4Δ/glk4Δ*	This work
*hxk2Δ/Δ glk1Δ/Δ glk4Δ/Δ*	*hxk2Δ*::FRT*/hxk2Δ*::FRT *glk1Δ/glk1Δ glk4Δ/glk4Δ*	This work
*hxk1Δ/Δ hxk2Δ/Δ glk1Δ/Δ glk4Δ/Δ*	*hxk1Δ/hxk1Δ hxk2Δ*::FRT*/hxk2Δ*::FRT *glk1Δ/glk1Δ glk4Δ/glk4Δ*	This work

### Growth Conditions and Media

Cells were grown at 30°C in SC medium supplemented with 2% glycerol, unless otherwise stated. SC medium contains 0.079% complete CSM (MP Biomedicals), 0.17% yeast nitrogen base without amino acids or ammonium sulfate (Difco), and 0.5% ammonium sulfate. During certain experiments, the cells were grown in YP medium which contains 1% yeast extract (Merck) and 2% bacteriological peptone (BD). During adhesion and biofilm experiments, RPMI 1640 with L-glutamine (Sigma) was used, which was buffered with 0.165 M morpholinepropanesulfonic acid (MOPS; Sigma) to pH 7. All media could be supplemented with 0.1%, 1%, or 2% glucose, fructose, galactose, glycerol, or GlcNAc depending on the experiment.

### Construction of Deletion Strains

The different deletion strains used in this study were made by two approaches: *SAT* Flipper method (Reuss et al., 2004) or a CRISPR genome editing method (Nguyen et al., 2017). The single deletion strains *hxk1Δ/Δ* and *hxk2Δ/Δ* were obtained via the *SAT* Flipper method (Reuss et al., 2004). For this, plasmid pSFS2 was used, which contains a reusable deletion cassette with a nourseothricin (NAT) marker; 500 bp upstream and downstream of the gene of interest was cloned upstream and downstream of this NAT cassette. Next, the plasmid was cut at the ApaI and SacI site, and the resulting fragment was transformed into the wild type (WT) strain to delete the first allele of the gene of interest. Deletion of the allele was checked via PCR. Cells with the correct mutation were grown on YP maltose (2%) to lose the NAT marker so that it could be reused for the deletion of the second allele. Other strains used in this study were made via the CRISPR genome editing system which could delete two alleles of a gene at the same time (Nguyen et al., 2017). A cloning free method was used to obtain different components of this system. Fragment A was amplified from plasmid pADH110 and contained the second part of the NAT marker. Fragment B was amplified from plasmid pADH147 and contained a specific gRNA. To obtain fragment C, fragments A and B were linked with each other via Phusion PCR. The *CAS 9* gene and the first part of the NAT marker were present on plasmid pADH99 which was cut at the MssI site to obtain a linear fragment. Both fragment C and the cut pADH99 plasmid were transformed into the strain of interest to delete the gene. The obtained colonies were checked via PCR and the ones with the deleted gene were cultured in YP maltose (2%) to lose the *CAS9* gene and the NAT marker. At least three independent transformants of each strain were created to work further with.

### Growth Curves

Strains were grown overnight in YP glycerol (2%). Cells were collected and washed twice with sterile Milli-Q water. Samples were diluted to OD_600_ 0.1 in sterile Milli-Q water; 20 μl of the cell suspension was added to 180 μl of SC medium supplemented with the indicated carbon source in a sterile 96-well plate. The OD_600_ was measured every half hour for 72 h in a Multiskan (Thermo Fisher).

### Kinase Activity

Cells were grown at 30°C in SC glycerol (2%). They were collected and washed twice with 1× imidazole buffer. The cell pellet was resuspended in 1× imidazole buffer (50 mM imidazole pH 7.5, 10 mM_Mgcl_2_, 100 mM KCl, and 0.1 mM EDTA) to a concentration of 100 mg cells/ml. Cells were lysed by fast prepping after the addition of glass beads and a protease inhibitor (complete, EDTA free; Roche) was added to prevent break down of proteins. The supernatant was collected and kinase activity was measured every 30 s for 5 min by making use of a test buffer which was specific for glucose or fructose. These test buffers contained 2× imidazole buffer, 50 mM ATP, 40 mM NADP, 0.6 U glucose-6-phosphate dehydrogenase (G6PDH; Sigma), 2 U phosphoglucose isomerase (PGI; only in the fructose buffer; Sigma), and 0.1% glucose or fructose. The protein concentration was measured by the Lowry method (Lowry et al., 1951).

### Gene Expression Analysis by qRT-PCR

Gene expression analysis was performed according to previously described method (Demuyser et al., 2017). The cells were grown to mid-exponential phase in SC medium supplemented with the carbon source of interest. Cells were washed with ice-cold Milli-Q water, frozen in liquid nitrogen and kept at −80°C. The cell pellet was dissolved in TRIzol (Thermo Fisher), and cells were lysed by fast prepping with glass beads. Afterward, RNA was isolated using chloroform, isopropanol, and 70% ethanol. The RNA was treated with DNase enzyme (New England Biolabs), to remove present DNA fractions, and converted into cDNA by using the iScript cDNA synthesis kit (Bio-Rad). To perform the quantitative PCR, a Go-Taq polymerase (Promega) and a StepOnePlus machine (Thermo Fisher) were used. The results were analyzed by making use of the qBasePlus software (Biogazelle).

### Glucose Transport

Cells were grown overnight at 30°C in SC glycerol (2%). Cells were collected, washed twice with MES buffer (10 mM; pH 6), and resuspended in SC medium to a concentration of 125 mg/ml. The dry weight of the cells was measured. Before the experiment, the cell suspension was incubated for 10 min at 30°C. Radioactive 0.05% ^14^C-glucose suspension (1500 cpm/nmol glucose; PerkinElmer) was added to the cells, and the mixture was incubated for 10 s. Afterward, uptake of glucose was blocked by adding ice-cold Milli-Q water to the cells. This solution was added on a filter to collect the cells. This filter was put in a scintillation tube with scintillation fluid (Lumagel Safe; PerkinElmer). The amount of radioactivity in the cells was measured with a scintillation counter (Hidex). Due to practicalities, only one biological transformant per mutant was tested during this experiment.

### Adhesion and Biofilm Formation

Cells were grown overnight in YP glycerol (2%). The cells were washed twice with 1× PBS and diluted in RPMI-MOPS (pH 7) to 1 × 10^7^ cells/ml; 100 μl of this cell suspension was added to a 96-well plate, which was precoated with FBS (Sigma). The plates were incubated at 37°C for 90 min. For adhesion, the medium was removed and 100 μl XTT solution (1 mg/ml; Fluka) was added and incubated for 1 h at 37°C. Afterward, the OD_490_ was measured. For biofilm formation, the medium was removed after 90 min at 37°C (adhesion), fresh RPMI medium was added, and the plate was put at 37°C for 24 h to obtain biofilm formation. The medium was removed, 100 μl XTT solution (1 mg/ml) was added, and the plate was incubated for 1 h at 37°C. The OD_490_ was measured.

### Gene Expression Analysis After Adhesion

Cells were collected after 90 min of adhesion in a six-well tissue culture to obtain the necessary number of cells. The wells were washed with 1× PBS and scraped off the bottom of the wells by making use of a sterile cell scraper. RNA was purified with the RiboPure-Yeast kit from Ambion. The RNA was treated with DNase enzyme (New England Biolabs), to remove present DNA fractions, and converted into cDNA by using the iScript cDNA synthesis kit (Bio-Rad). To perform the quantitative PCR, a Go-Taq polymerase (Promega) and a StepOnePlus machine (Thermo Fisher) were used. The results were analyzed by making use of the qBasePlus software (Biogazelle).

### Cultivation of Primary Bone Marrow-Derived Macrophages

The cultivation of primary bone marrow-derived macrophages (BMDMs) was performed as described previously (Riedelberger et al., 2020). Briefly, bone marrow was isolated from femur and tibia of C57BL/6J mice and cultivated for 10 days in BMDM medium [Dulbecco’s modified Eagle’s medium (DMEM; Gibco), 10% heat-inactivated FCS (Sigma), 100 U/ml penicillin, 100 μg/ml streptomycin and L-conditioned media)]. BMDMs were harvested and split into the respective cell culture dishes 1 day before the experiment.

### *In vitro C. albicans* Survival Experiment

The *in vitro C. albicans* experiment was performed as previously described with minor modifications (Riedelberger et al., 2020). BMDMs were seeded in a 96-well plate (1 × 10^5^ cells/well) and infected with *C. albicans* cells (1 × 10^4^ cells/well) in triplicates. This co-culture was incubated for 3 h at 37°C. Afterward, 4% Triton-X 100/PBS solution was added to the cells. The *C. albicans* cells were collected and plated, and colony forming units (CFU) were counted. Due to practicalities, only one biological transformant per mutant was tested during this experiment.

### Disseminated Infection

One week prior to the injection, 8 weeks old female BALB/c mice (Janvier) were randomly taken from the transport box and put in their cage. Animals were housed in groups of four per cage, and food and water were provided *ad libitum*. Cotton pads, wood sticks, and paper rolls were placed in the cages. Day/night regime was applied, and temperature and humidity were monitored every day. *C. albicans* cells were grown overnight in YP glycerol (2%). They were collected and washed with 1× PBS. The cells were diluted to a final concentration of 3.75 × 10^6^ cells/ml; 200 μl of this inoculum was injected in the lateral tail vein and the inoculum was verified via CFU counting. For the survival experiment, mice were checked for 20 days and killed when humane endpoints were reached. These humane endpoints included weight loss by decreased food and water uptake, hunched posture, lethargy and ruffled fur. Due to practicalities, only one biological transformant per mutant was tested during this experiment. The sample size was calculated with *f* = 0.5 and *p* = 0.8 so each group consisted out of 8 mice. To check the dissemination of *C. albicans* cells in the kidneys, infected mice were sacrificed 3 days post-injection. The kidneys were collected, homogenized, and plated to count CFUs. Due to practicalities, only one biological transformant per mutant was tested during this experiment. The sample size was calculated with *f* = 0.8 and *p* = 0.8 so each group consisted out of 4 mice.

### Ethics Statement

The animal experiments were approved by the ethical committee of KU Leuven (project number P023/2019) and were performed in accordance with the regulations of the KU Leuven animal care guidelines. The KU Leuven ethical committee for animal experimentation is part of the regional regulations imposed by the Flemish government, Secretary General of the Unit Animal Welfare, “Departement Leefmilieu, Natuur & Energie,” Koning Albert II-laan 20 bus 8, 1000 Brussel. All mice were kept in filter-top cages in an animal room with adjusted temperature, humidity, and light.

## Results

### Deletion of Sugar Kinases Affects the Growth on Glucose and Fructose

To investigate the roles of different sugar kinases for growth on different carbon sources, we generated the respective single and multiple deletion strains using the *SAT* flipper cassette (Reuss et al., 2004) or the CRISPR Cas9 system (Nguyen et al., 2017). We were able to generate seven different deletion strains: *hxk1Δ/Δ, hxk2Δ/Δ*, hxk1Δ/Δ *hxk2Δ/Δ*, *glk1Δ/Δ glk4Δ/Δ*, *hxk1Δ/Δ glk1Δ/Δ glk4Δ/Δ*, *hxk2Δ/Δ glk1Δ/Δ glk4Δ/Δ* and the *hxk1Δ/Δ hxk2Δ/Δ glk1Δ/Δ glk4Δ/Δ* strain. For clarity, we will refer to them without the delta symbol from here onward. We were not able to generate single deletion strains of *GLK1* and *GLK4* because they were 98% homologous. Therefore, it was not possible to delete the genes separately, for which we will refer to these enzymes as Glk1/4.

**FIGURE 1:**
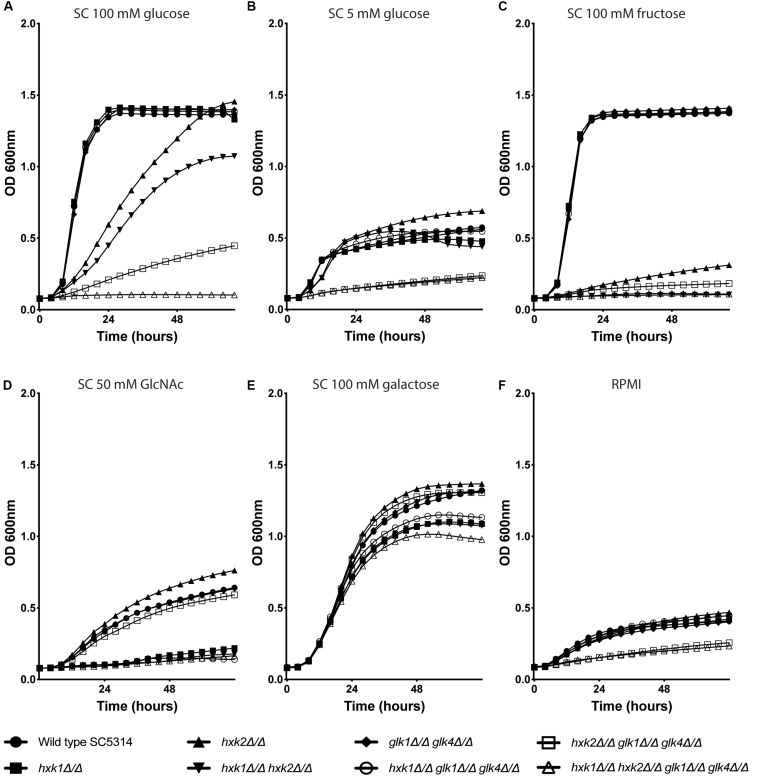
Hxk2 is important for the growth on glucose and fructose. The different strains were grown in different media: SC medium supplemented with 2% glucose **(A)**, 0.1% glucose **(B)**, 2% fructose **(C)**, 1% GlcNAc **(D)**, 2% galactose **(E),** and RPMI medium **(F)**. The OD_600_ was followed over time for 72 h. WT(SC5314) (•), *hxk1* (■), *hxk2* (▲), *hxk1 hxk2* (▼), *glk1 glk4* (◆), *hxk1 glk1 glk4* (○), *hxk2 glk1 glk4* (□), and *hxk1 hxk2 glk1 glk4* (△). The data represent the average of two independent experiments.

To check the role of individual and multiple kinases on growth, the different strains were grown in RPMI medium or in SC medium supplemented with 0.1% glucose, 2% glucose, 2% fructose, 2% galactose, or 1% GlcNAc (Figure 1). We hypothesized that the deletion of one or multiple kinases would affect the growth of *C. albicans* on glucose and fructose containing media, due to a possible reduced glycolytic flux. For growth on 2% of glucose, *hxk2, hxk1 hxk2* and *hxk2 glk1 glk4* showed a reduced growth compared to the WT (Figure 1A). Moreover, the quadruple deletion strain, *hxk1 hxk2 glk1 glk4*, was not able to grow on this medium, indicating that all four kinases play a role during growth on high glucose concentrations. Hxk2 was sufficient for full growth on glucose containing medium but also strains expressing only *HXK1* were able to grow on medium containing high glucose levels (Figure 1A), which is in line with previous studies (Naseem et al., 2011). This was also visible in the growth curves on SC 1% GlcNAc (Figure 1D). Strains which lacked the *HXK1* gene (*hxk1*, *hxk1 hxk2*, *hxk1 glk1 glk4*, and *hxk1 hxk2 glk1 glk4*) were not able to grow at the same rate on this carbon source as the WT. In RPMI (which contains 0.2% glucose) or SC supplemented with 0.1% of glucose, a similar reduced growth was seen for *hxk2 glk1 glk4* and *hxk1 hxk2 glk1 glk4*. The difference in growth rate between the WT and the two mutant strains was less pronounced than in the presence of 2% glucose, as all strains grew slower at low glucose levels (Figures 1B,F). In contrast, for growth on 2% fructose, both *HXK1* and *HXK2* were important. The *hxk2* mutant showed a strongly reduced growth, while the double deletion strain *hxk1 hxk2* was not able to grow on 2% fructose (Figure 1C). This indicates that Hxk2 is the most important kinase for growth on fructose, while Hxk1 seems to have a limited role. When 2% galactose was used as a carbon source, the initial growth was the same for all strains (Figure 1E), indicating that uptake and metabolism of galactose is similar for all strains. Finally, the growth properties of different strains were confirmed by spot assays on media containing different carbon sources (data not shown). As a control, we used SC 2% glycerol as a carbon source, and no difference in growth was observed for any of the strains (data not shown).

Taken together, the growth curves suggested that all four kinases are important for growth under high glucose conditions. During low glucose levels, Hxk1 had no effect on fungal growth. Upon high fructose concentrations, Hxk2 emerges as the major kinase for growth, with a minor role for Hxk1.

### Glucose Phosphorylation Depends on Hxk2 and Glk1/4

**FIGURE 2:**
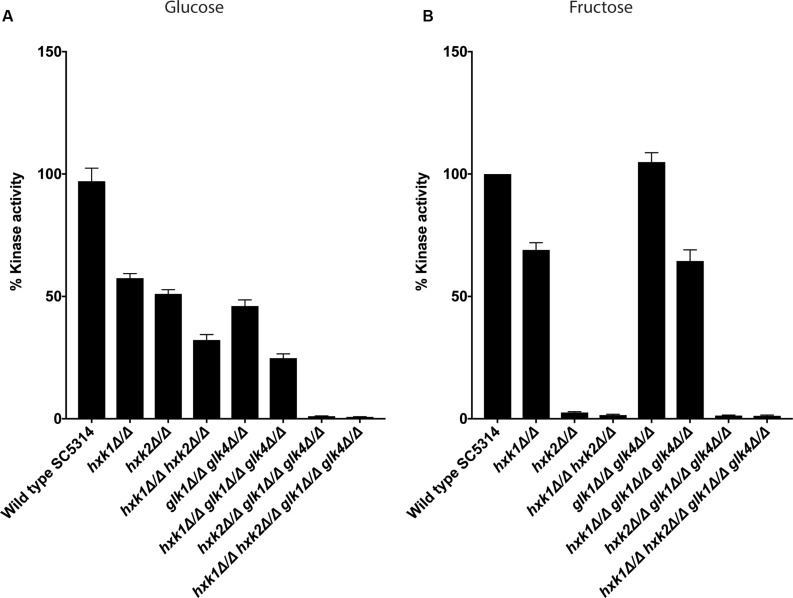
Both Hxk2 and Glk1/4 play a role in glucose metabolism, while only Hxk2 has an effect on fructose metabolism. The cells were grown in SC medium with 2% glycerol before proteins were isolated and kinase activity was measured. The values of the deletion strains are normalized relative to the wild type strain which has 100% kinase activity. **(A)** Kinase activity on glucose. **(B)** Kinase activity on fructose. The average of three independent experiments is shown, and the error bars represent the standard error of the mean (SEM).

The growth assays indicated that different kinases were important for growth on glucose and fructose containing media. Therefore, we determined the enzymatic activity of the different kinases during glucose or fructose phosphorylation. In *S. cerevisiae*, *Sc*Hxk1, *Sc*Hxk2, and *Sc*Glk1 convert glucose into glucose-6-phosphate, while *Sc*Hxk1 and *Sc*Hxk2 phosphorylate fructose (Herrero et al., 1995). Since this experiment was performed under low sugar concentrations we hypothesized, based on growth data (Figure 1), that Hxk2 and Glk1/4 would play a role in glucose phosphorylation, while Hxk2, and to a lesser extent Hxk1, would mediate fructose phosphorylation. Strikingly, Hxk2 and Glk1/4 were important for glucose phosphorylation, since kinase deletion reduced activity of around 50% and the triple mutant *hxk2 glk1 glk4* showed no kinase activity (Figure 2A). This also indicated that Hxk1 lacked glucose phosphorylating activity under the tested conditions. However, deletion of only *HXK1* reduced kinase activity to 60% of total activity (Figure 2A). Hence, Hxk1 presumably regulated the expression and/or activity of other sugar kinases. In contrast, fructose was only metabolized by Hxk2, as all strains lacking *HXK2* were unable to phosphorylate this sugar (Figure 2B). For Hxk1, similar results as for glucose were found, showing that Hxk1 deletion reduced the total kinase activity, whereas this kinase cannot phosphorylate fructose. This strengthened our hypothesis concerning a possible regulatory function for Hxk1 in the presence of glucose or fructose.

### Hxk1 Regulates the Expression of *HXK2* and *GLK1/4*

**FIGURE 3:**
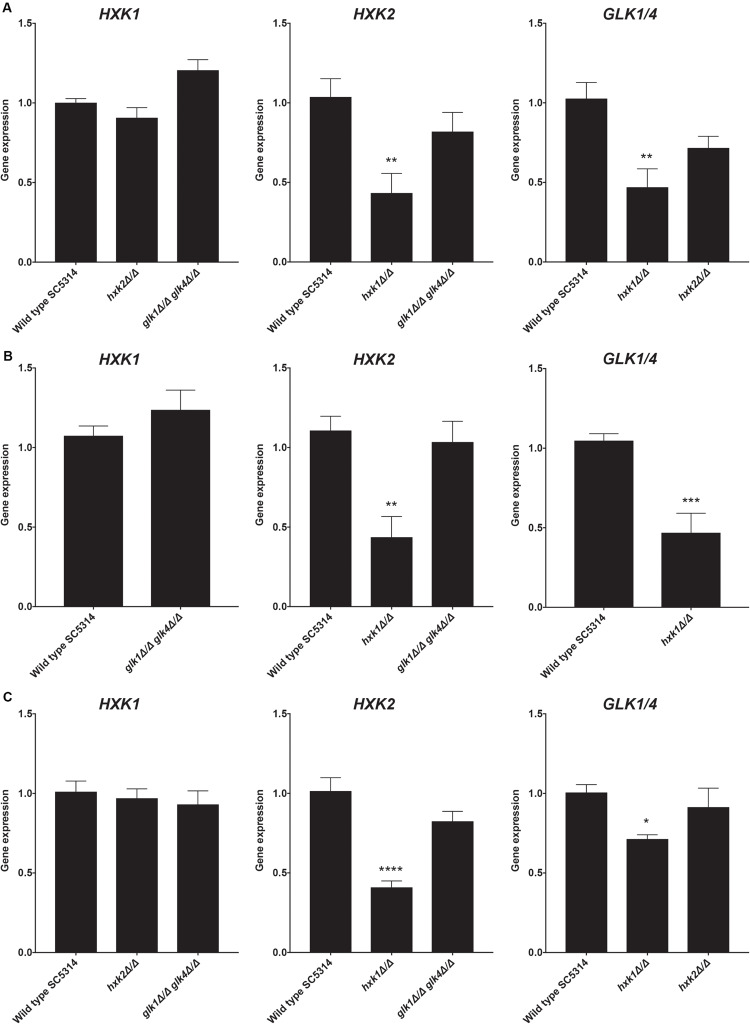
Deletion of *HXK1* causes a decreased expression of *HXK2* and *GLK1/4*. Cells were grown at 30°C in SC medium supplemented with a specific carbon source. RT-qPCR was used to study gene expression. **(A)** Expression levels of the different genes when cells were grown on SC with 2% glucose. **(B)** Expression levels of the different genes when cells were grown on SC with 2% fructose. **(C)** Expression levels of the different genes when cells were grown on SC with 2% glycerol. The average of two independent experiments is shown and the error bars represent the SEM. Statistical analysis was done on log_2_(y)-transformed values by using an ANOVA test with Bonferroni correction. For the expression of *HXK1* and *GLK1/4* on fructose, a *t*-test was used. A significant difference was found when *p = 0.0332, **p = 0.0021, ***p = 0.0002 or ****p < 0.0001.

The *hxk1* mutant showed less glucose and fructose phosphorylating activity when compared to the WT strain, while Hxk1 had no activity by itself. Hence, we hypothesized that deleting *HXK1* would lead to a reduced expression of *HXK2* and *GLK1/4* on either glucose or fructose. Indeed, upon deletion of *HXK1*, the expression of *HXK2* and *GLK1/4* was downregulated in both glucose- and fructose-containing media (Figures 3A,B). Furthermore, this regulation seemed to be carbon source-independent, as a similar regulation was observed in cells grown on glycerol (Figure 3C). These results supported our notion that Hxk1 could regulate the expression of *HXK2* and *GLK1/4* in a positive manner.

As a control, we also performed the reverse experiment, whereby *HXK2* deletion did not alter the expression of other sugar kinases (Figure 3). In contrast, as part of the glucose repression system, deletion of *ScHXK2* strongly affects the gene expression of other sugar kinases in *S. cerevisiae* (Rodriguez et al., 2001) genes. Deletion of *GLK1/4* did also not alter the expression of the other kinase genes. These results showed the difference in glucose repression pathways between *S. cerevisiae* and *C. albicans.*

### Deletion of *HXK1* or *HXK2* Results in an Increased Glucose Transport Capacity

**FIGURE 4:**
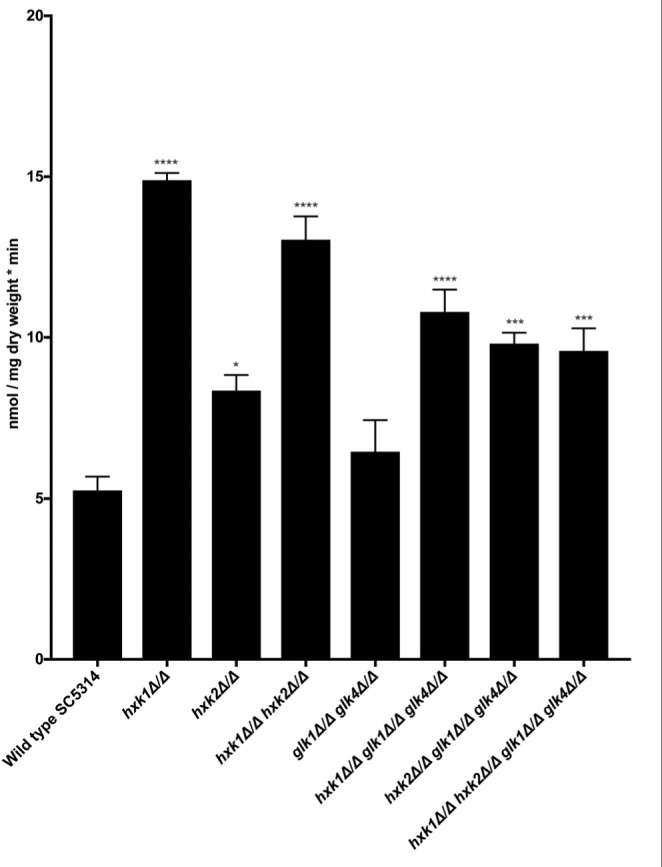
Hxk1 and Hxk2 seem to play a role in glucose transport. A radioactive 0.05% glucose suspension containing ^14^C (1500 cpm/nmol glucose) was added to the different mutant strains and the amount of radioactive glucose was measured. The average of three independent experiments is shown and the error bars represent the SEM. Statistical analysis was done by using an ANOVA test with Bonferroni correction. A significant difference was found when *p = 0.0332, ***p = 0.0002 or ****p < 0.0001.

In *C. albicans*, glucose transporter genes are regulated by the glucose repression system, including Rgt1, Std1, Mig1, and Mig2 (Sexton et al., 2007; Lagree et al., 2020). In fact, deletion of *HXK2* increased the expression of glucose transporter genes *HGT7*, *HGT12*, and *HXT10* (Laurian et al., 2019). Therefore, we hypothesized that a deletion of *HXK2* would increase glucose uptake when compared to the WT, as the *hxk2* mutant would result in higher expression of the different glucose transporter genes (Laurian et al., 2019). Indeed, when glucose uptake was measured, deletion of *HXK2* caused significantly higher glucose transport rates than the WT strain (Figure 4). By contrast, deletion of *GLK1/4* did not affect glucose uptake (Figure 4). These results supported earlier reports, showing that deletion of *HXK2* caused an increased expression of glucose transporter genes which explained higher glucose transport rates (Laurian et al., 2019). Furthermore, the results also suggested a possible direct or indirect (via decreased *HXK2* expression) role for Hxk1 in glucose transport.

### A Disrupted Glycolytic Pathway Affects the Adhesion Capacity of *C. albicans*

**FIGURE 5:**
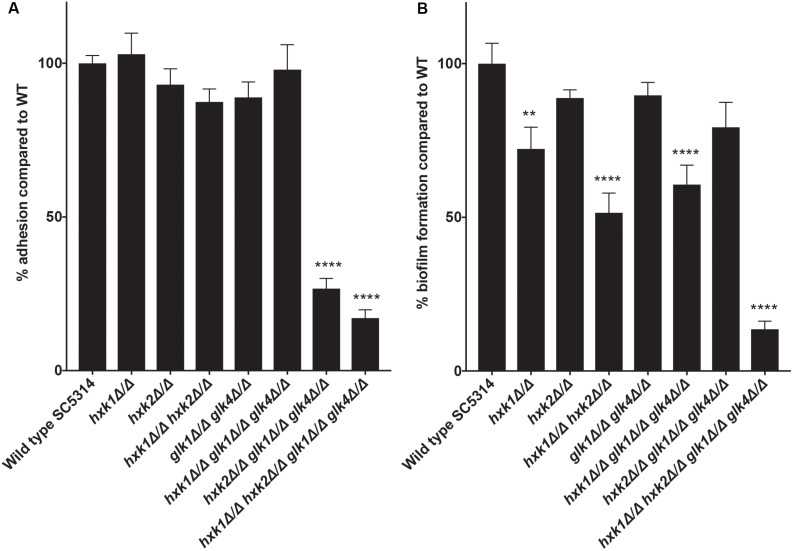
**(A)** A disrupted glycolytic pathway has an effect on the adhesion capacity of *C. albicans*. The adhesion capacity was measured after 90 minutes by using XTT. The values of the deletion strains are normalized relative to the WT strain which has 100% adhesion capacity. The results show the average of three independent experiments with the error bars representing the SEM. Statistical analysis was done by using an ANOVA test with Bonferroni correction. A significant difference was found when ****p < 0.0001. **(B)** Deletion of *HXK1* causes a decreased biofilm formation. The biofilm formation capacity was measured after 24 hours by using XTT. The values of the deletion strains are normalized relative to the WT strain which has 100% biofilm formation capacity. The results show the average of three independent experiments with the error bars representing the SEM. Statistical analysis was done by using an ANOVA test with Bonferroni correction. A significant difference was found when **p = 0.0021 or ****p < 0.0001.

**FIGURE 6:**
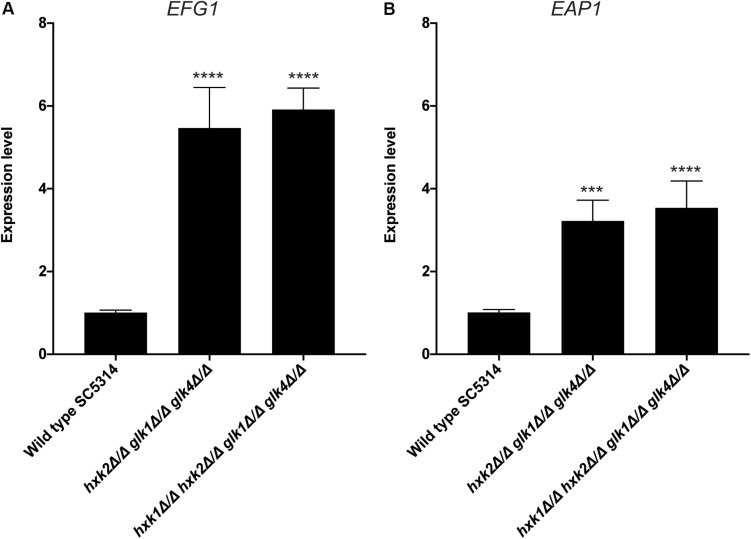
Deletion of *HXK2* and *GLK1/4* causes an increased expression of *EFG1* and *EAP1*. Cells were adhered for 90 minutes. Afterwards, adhered cells were collected and RT-qPCR was used to study gene expression. **(A)** The expression levels of *EFG1*. **(B)** The expression levels of *EAP1*. The average of two independent experiments is shown and the error bars represent the SEM. Statistical analysis was done on log_2_(y)-transformed values by using an ANOVA test with Bonferroni correction. A significant difference was found when ***p = 0.0002 or ****p < 0.0001.

An important virulence factor of *C. albicans* is its adhesion capacity, leading to the formation of biofilms, which are frequently resistant against available antifungal drugs (Seneviratne et al., 2008; Řičicová et al., 2010; Sanglard, 2019). Therefore, we tested the mutant strains for *in vitro* adhesion and biofilm formation to polystyrene plates coated with serum. To mimic a physiological environment, we cultured fungal cells in RPMI-1640 medium. For adhesion, *hxk2 glk1 glk4* and *hxk1 hxk2 glk1 glk4* showed a reduced adhesion capacity when compared to the WT strain (Figure 5A). As cells were only incubated for 90 min, this low adhesion phenotype was independent of the slow growth in RPMI medium (Figure 1F). To determine whether the lower adhesion was due to altered gene expression of known proteins important for this phenotype, RT-qPCR analysis was performed after 90 min of adhesion. Thereby, the expression of *EFG1* and *EAP1* was upregulated in the *hxk2 glk1 glk4* and *hxk1 hxk2 glk1 glk4* strain (Figure 6) which was rather unexpected as the expression of these genes favors the adhesion of *C. albicans* (Sonneborn et al., 2000; Li and Palecek, 2003).

In the biofilm experiment, strains with a deletion in *HXK1* showed a reduced ability to form biofilms (Figure 5B). This was possibly due to the hyperfilamentation phenotype of these strains caused by a *HXK1* deletion, as previously reported (Naseem et al., 2011). All other strains, including the *hxk2 glk1 glk4* strain, with a reduced adhesion, showed no significant difference in biofilm formation. Taken together, glycolytic flux is important during adhesion, while it has no effect on biofilm formation.

### The Sugar Kinases Do Not Affect *C. albicans* Survival in Macrophages *in vitro*

**FIGURE 7:**
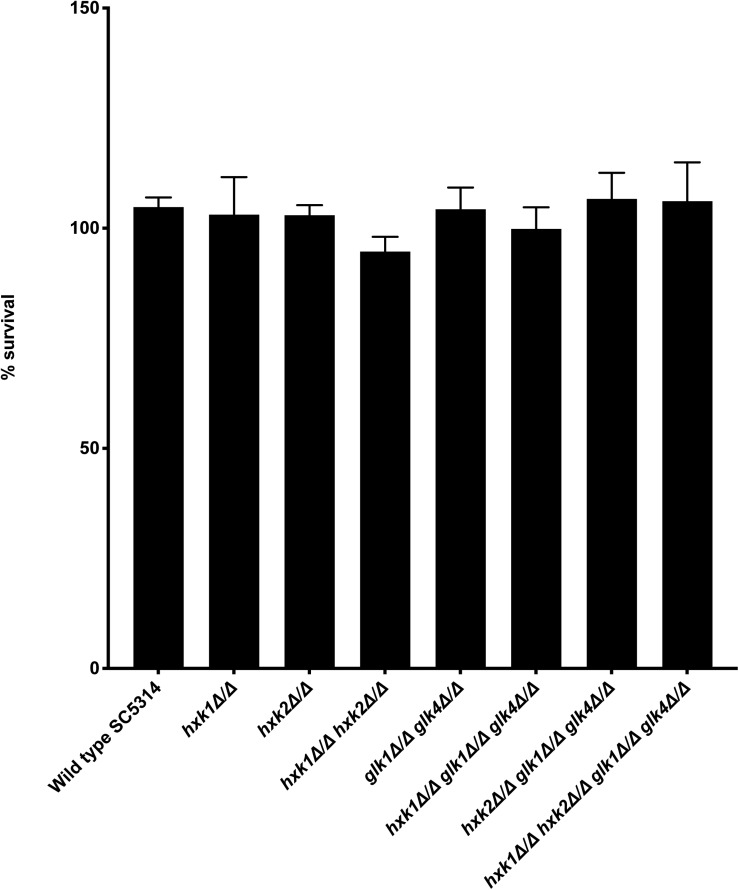
No difference is observed for survival between WT and mutants when co-cultured with BMDMs. *C. albicans* cells were co-cultured for 3 h with BMDMs at 37°C. Afterward, the fungal cells were plated to check for their survival. The results shown are the average of three independent experiments with the error bars representing the SEM.

Various immune surveillance mechanisms impair the systemic dissemination of *C. albicans* upon host invasion, whereby innate immune cells such as neutrophils and macrophages are crucial for antifungal immunity (Netea et al., 2015). Thus, we investigated the role of sugar kinases on the survival of *C. albicans* upon infection with primary BMDMs. No survival difference between WT and mutants was observed upon co-culture with BMDMs (Figure 7). Hence, at least under the tested conditions, sugar phosphorylation is dispensable for *C. albicans* survival macrophage infection.

### Glycolysis Controls Virulence of *C. albicans* During Systemic Infections

**FIGURE 8:**
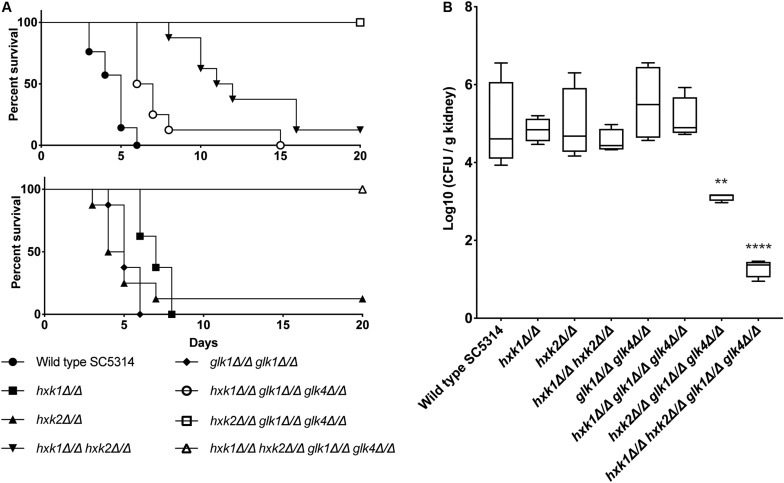
**(A)** *HXK2* and *GLK1/4* regulate fungal virulence of *C. albicans* in a mouse model for systemic infection. The strains were injected in the lateral tail vein of Balb/c mice with a final concentration of 7.5 × 10^5^ cells per injection. The infected mice were checked two times a day for 20 days. When humane endpoints were reached, mice were killed. Statistical analysis was done by using a log rank test and a significant difference was found when ****p < 0.0001. WT(SC5314) (•), *hxk1* (■)^****^, *hxk2* (▲), *hxk1 hxk2* (▼)^****^, *glk1 glk4* (◆), *hxk1 glk1 glk4* (○)^****^, *hxk2 glk1 glk4* (□)^****^, *hxk1 hxk2 glk1 glk4*(△)^****^. **(B)** *hxk2 glk1 glk4* and *hxk1 hxk2 glk1 glk4* show significant less kidney colonization compared to the wild type. Cells were injected in the lateral tail vein of Balb/c mice with a final concentration of 7.5 × 10^5^ cells per injection. Three days post-injection, mice were killed and kidneys were collected. The amount of *C. albicans* cells was verified via CFU counting. The boxplots represent the average log10 (CFU/g kidney) of the different strains tested. Statistical analysis was done by using an ANOVA test with Bonferroni correction. A significant difference was found when **p = 0.0021 or ****p < 0.0001.

We tested the different mutant strains in a mouse model for systemic infection to see the effect of the deletions in an *in vivo* setting. Strikingly, all strains lacking *HXK1* (*hxk1*, *hxk1 hxk2, hxk1 glk1 glk4*, and *hxk1 hxk2 glk1 glk4*) showed a significant impaired virulence compared to the WT strain (Figure 8A). The *hxk2 glk1 glk4* and *hxk1 hxk2 glk1 glk4* mutant strains were completely avirulent during a systemic infection (Figure 8A). The *hxk2* and *glk1 glk4* mutant strains were as virulent as the WT strain. Additionally, we also determined fungal kidney loads 3 days post-infection. This time point was chosen since animals, infected with WT *C. albicans*, succumbed to systemic infection starting at day 3. Interestingly, in line with their avirulent phenotypes, the *hxk2 glk1 glk4* and *hxk1 hxk2 glk1 glk4* strains revealed reduced fungal burden in murine kidneys (Figure 8B). In contrast, all other strains did not show significant differences in kidney colonization when compared to the WT. Owing to the survival data and the identical kidney loads during the initial infection phase, we conclude that animals infected with *hxk1 hxk2 or hxk1 glk1 glk4* can better cope with kidney invasion, which is otherwise lethal for WT strain-infected mice. Taken together, these data demonstrate that a functional glycolytic flux is central for maximal fungal virulence during systemic candidiasis.

## Discussion

In this study, we demonstrate that sugar kinases of *C. albicans* are important for glycolytic sugar metabolism and virulence. After sequence alignment with *S. cerevisiae*, we identify four putative kinases: Hxk1, Hxk2, Glk1, and Glk4. As the latter two enzymes share 98% gene identity, it is not possible to delete these genes separately for which we will refer to these enzymes as Glk1/4. We show that Hxk2 and Glk1/4 are important for glucose metabolism, while only Hxk2 plays a role in fructose phosphorylation. Additionally, we identify a regulatory role for Hxk1, which controls the expression of *HXK2* and *GLK1/4* in a positive manner. When *HXK1* is deleted, the expression level of the other kinases is drastically reduced, and strains with a *HXK1* deletion shows a lower biofilm forming capacity. During this study, another report suggests a role for Hxk2, Glk1, and Glk4 in sugar phosphorylation, while Hxk1 is not included in the study (Laurian et al., 2019). We show that Hxk1 has profound effects on carbon source utilization in glucose and fructose containing medium and that its presence affects the expression of the other sugar kinases.

Under high glucose conditions, Hxk2 is the most important kinase for growth, since a *hxk2* mutant shows a decreased proliferation. Likewise, Glk1/4 also contributes to growth on high sugar concentrations, but is functionally redundant due to Hxk2. A *glk1 glk4* mutant shows the same growth capacity as a WT, while a mutant strain with only *GLK1/4* present is still able to grow on glucose albeit at a lower rate. In the presence of low glucose, this functional redundancy of Glk1/4 by Hxk2 is absent, because a single deletion of the kinases phenocopies the growth phenotype of the WT strain. This is consistent with earlier reports, showing that a *glk1 glk4* mutant retains full kinase activity, while a *hxk2* mutant loses 60% of its activity when high glucose levels are present (Laurian et al., 2019). During our kinase activity experiments, we use low (physiological) glucose concentrations, explaining that we do not see this functional redundancy effect of Glk1/4 by Hxk2. A deletion of one of these enzymes causes a 50% reduction in kinase activity, while deletion of both *HXK2* and *GLK1/4* impairs growth on low glucose concentrations. Furthermore, a *hxk2 glk1 glk4* mutant and a *hxk1 hxk2 glk1 glk4* mutant show similar growth rates, suggesting that only Hxk2 and Glk1/4 have a role in the presence of low glucose concentrations. This is confirmed by kinase experiments, where no activity is measured for the *hxk2 glk1 glk4* and *hxk1 hxk2 glk1 glk4* mutant strains. Further, Hxk2 has no effect on the gene expression of other sugar kinases in *C. albicans* which is the case for *S. cerevisiae* (Rodriguez et al., 2001). This shows that this part of the glucose repression system works differently in *C. albicans* and *S. cerevisiae*. Presumably, the regulatory function of *Sc*Hxk2 is partially taken over by Hxk1 in *C. albicans*, as we observed a rather regulatory function for Hxk1.

The fructose growth data suggest that both Hxk2 and, to a lesser extent, Hxk1 play a role in fructose metabolism. However, the kinase activities hint that only Hxk2 is involved in fructose phosphorylation. When *HXK2* is deleted, but *HXK1* is still present, fructose kinase activity is completely lost, confirming previous reports (Laurian et al., 2019).

The role of Hxk1 is rather peculiar in this story. Hxk1 is important in GlcNAc metabolism, but we also demonstrate a novel function in glucose and fructose metabolism (Kumar et al., 2000). We show that a strain harboring only *HXK1* still grows slowly on glucose and fructose, although Hxk1 shows no sugar kinase activity. The fungal growth cannot be attributed to the availability of GlcNAc, because this component is not present in the medium. How a mutant strain with only this single kinase can grow remains unclear, but possibly, *HXK1* induces expression or activates enzymes involved in the metabolism of alternative carbon sources such as amino acids. In this way, *C. albicans* can still grow due to the consumption of alternative carbon sources. We have also identified a regulatory role for Hxk1 on the expression of *HXK2* and *GLK1/4*. When *HXK1* is deleted, the other kinases show diminished expression. Our results show that Hxk1 has a positive (direct or indirect) effect on the gene expression of other sugar kinases, and thereby, Hxk1 is important for an efficient glucose and fructose phosphorylation.

Although the *hxk1 hxk2 glk1 glk4* mutant slowly grows at SC 0.1% glucose, it is unable to grow on SC 2% glucose, because it cannot phosphorylate glucose. This suggests an involved alternative pathway for amino acids utilization in the absence of glucose. Our hypothesis is that this pathway is blocked in the presence of high glucose concentrations, but not in the presence of low glucose. This might be an explanation for the quadruple mutant, which slowly grows in SC 0.1% glucose, but does not grow in SC 2% glucose. Further, the putative involvement of alternative metabolic pathways for carbon source utilization might be an absorbing scientific question for future studies.

The transport data demonstrate a role for *HXK1* and *HXK2* during glucose transport in *C. albicans*. A deletion of *HXK2* increases glucose transport, confirming earlier reports indicating that Hxk2 enters the nucleus when 0.1% glucose is present and thereby has a negative effect on the expression of glucose transporter genes. A deletion of *HXK2* increases the expression of glucose transporter genes *HGT7*, *HGT12*, and *HXT10* (Laurian et al., 2019). Our findings clearly support the hypothesis of Laurian and coworkers that Hxk2 represses glucose transporter gene expression and thereby reduces glucose uptake (Laurian et al., 2019). In contrast, the role of Hxk1 remains rather unclear. It is possible that Hxk1 has a direct effect on glucose transport via a similar mechanism as Hxk2. Of note, Hxk1 also migrates to the nucleus when glucose is present (Rao et al., 2014). Therefore, Hxk1 might block the expression of defined glucose transporter genes by transcriptional repression. However, it is also plausible that Hxk1 has an indirect effect on glucose transport. RT-qPCR analysis revealed that Hxk1 enhances *HXK2* expression. Thus, *HXK1* deletion reduces *HXK2* expression, which might lead to an increased expression of glucose transporter genes, leading to elevated glucose uptake.

Diverse macrophage subsets are key players in the innate immune defense against *C. albicans* (Traven and Naderer, 2019). However, *C. albicans* counteracts macrophage defenses leading to immune cell destruction (Uwamahoro et al., 2014; Wellington et al., 2014; Kasper et al., 2018). During the early stages of macrophage interaction, alternative carbon utilization pathways such as the glyoxylate cycle, gluconeogenesis, and fatty-acid ß-oxidation are important (Lorenz and Fink, 2001, 2002; Lorenz et al., 2004; Ramirez and Lorenz, 2007). Alternative carbon sources, like GlcNAc, are important for counteracting the antifungal activity exerted by macrophages (Williams and Lorenz, 2020). GlcNAc catabolism neutralizes the pH of the phagolysosomes, and thereby, it helps *C. albicans* to destruct the immune cells (Vesely et al., 2017). A strain with the deletion of three genes involved in this GlcNAc catabolism (*HXK1*, *DAC1*, and *NAG1*) is less capable of destructing macrophages upon coculture (Vesely et al., 2017; Williams and Lorenz, 2020). However, we did not observe a reduction in *C. albicans* survival upon deletion of *HXK1*. Probably, all three enzymes need to be deleted to have a reduced virulence in the macrophage assay. At later stages of the interaction, the role of glucose becomes important (Traven and Naderer, 2019). During these later stage host–pathogen interactions, glycolytic genes are upregulated in both macrophages and fungal cells, owing to the metabolic stress *C. albicans* encounters after phagocytosis, leading to increased fungal glucose import. Interestingly, *HXK2* is only upregulated during advanced macrophage infections (Tucey et al., 2018). These data are in line with our macrophage experiment, where we do not see any survival difference of fungal strains after 3 h of BMDM infection, showing that sugar kinases are dispensable during the early stage of BMDM infections.

We show that the *hxk1 glk1 glk4* and *hxk1 hxk2 glk1 glk4* deletion strains display defects in adhesion. Possibly, this is due to a decreased glycolytic flux, since *C. albicans* adheres stronger in the presence of glucose (Yin et al., 2004). However, it is also possible that other mechanisms are involved in this adhesion defect, such as masking specific adhesins. Since low glucose levels are present, alternative carbon sources could play an important role. For example, catabolite inactivation is slow in *C. albicans*, leading to prolonged use of alternative carbon sources and causing metabolic flexibility which is critical to colonize different niches in the body (Sandai et al., 2012). Furthermore, different alternative carbon utilization pathways, like gluconeogenesis, glyoxylate cycle, and fatty-acid ß-oxidation, are important during infection and immune response, as previously mentioned (Lorenz and Fink, 2001, 2002; Lorenz et al., 2004; Ramirez and Lorenz, 2007). Taken together, our data support the importance of flexible carbon utilization during infection, although further research is necessary to elucidate the role of glucose flux and alternative carbon sources in this hexokinase-dependent adhesion.

*EFG1* and *EAP1* are two genes with a positive effect on the adhesion of *C. albicans*. Efg1 is a transcription factor which is phosphorylated by PKA and initiates the expression of *EAP1* (Sonneborn et al., 2000; Li and Palecek, 2003). We observe that strains with less adhesion capacity (*hxk2 glk1 glk4* and *hxk1 hxk2 glk1 glk4*) have a higher expression of *EFG1* and *EAP1*. This is rather unexpected, since deletion of *EFG1* or *EAP1* reduces adhesion, while *EAP1* overexpression increases adhesion (Li and Palecek, 2003; Li et al., 2007). Efg1 autoregulates its own expression level, which is even more increased in the phosphorylated protein state (Tebarth et al., 2003). Therefore, it is possible that Efg1 is not phosphorylated by PKA in the *hxk2 glk1 glk4* and *hxk1 hxk2 glk1 glk4* strains, implying that the *EFG1* expression is not inhibited anymore. Since Efg1 is not phosphorylated by PKA, it cannot activate its downstream genes, which causes the adhesion of *C. albicans*. In this way, it is possible that both a higher expression level of *EFG1* and less adhesion is observed in these mutant strains. However, this does not explain why also a higher expression of *EAP1* is observed. Another explanation for these findings can be that overexpression of *EFG1* causes only pseudohyphae formation (Stoldt et al., 1997). It is known that pseudohyphae adhere less to oral keratinocytes compared to true hyphae, while they are still able to form biofilms (Villar et al., 2004; Lopez-Ribot, 2005). Therefore, the decreased adhesion observed in the *hxk2 glk1 glk4 and hxk1 hxk2 glk1 glk4* mutants might be caused by the formation of pseudohyphae due to *EFG1* overexpression. This might also explain the normal biofilm formation of *hxk2 glk1 glk4*, although its adhesion capacity is drastically reduced.

Biofilm formation seems to work independently from glucose metabolism. The strain with a disrupted glycolytic pathway, *hxk2 glk1 glk4*, is able to form biofilms just like the WT strain. On the other hand, Hxk1 appears to be important for biofilm formation. The deletion of *HXK1* causes significant less biofilm formation than the WT. This is unexpected, because we assumed that these strains would form thicker biofilms due to their hyperfilamentation phenotype (Singh et al., 2001; Naseem et al., 2011). However, it is known that Hxk1 interacts with the histone deacetylase Sir2 under hyphal inducing conditions and thereby controls gene expression (Rao et al., 2013). It is plausible that Hxk1 has a positive effect on the expression of biofilm inducing genes via this mechanism and that due to a *HXK1* deletion, a decreased biofilm formation occurs.

The *hxk2* mutant has a defect in hyphal formation on media with specific carbon sources that need to be phosphorylated by Hxk2, and this defect is slightly worsened by deletion of *GLK1*. Therefore, the hyphal defect seems linked with a defect in sugar phosphorylation (Laurian et al., 2019). Furthermore, a higher survival of *Galleria mellonella* larvae upon infection with a *hxk2* or *hxk2 glk1* mutant is observed when compared to the WT. Therefore, attenuated virulence could be due to the hyphal defect, as *C. albicans* is unable to invade and penetrate tissue (Laurian et al., 2019). We show that the *hxk1 glk1 glk4* and *hxk1 hxk2 glk1 glk4* deletion strains have an attenuated virulence during systemic infections in mice. However, we think that this reduced virulence is not due to the filamentation defect upon deletion of *HXK2* observed in Laurian et al. If the hypothesis of Laurian and coworkers about the filamentation defect and glucose phosphorylation is true, a *hxk2* mutant should be less virulent in a systemic infection, but this is not the case in our study. Therefore, we think that this reduction in virulence is not due to the hyphal defect, but rather a consequence of decreased glycolytic flux. Glycolysis is upregulated when *C. albicans* faces immune defense (Barelle et al., 2006). It was also demonstrated that deletion of activators of glycolytic enzymes causes attenuated virulence, and gluconeogenic genes and the glyoxylate cycle are inactive in the presence of physiological relevant glucose concentrations, e.g., during kidney infections (Barelle et al., 2006; Askew et al., 2009). Therefore, we propose that glycolysis plays a major role during kidney invasion and by blocking the glycolytic flux strongly impairs fitness and virulence of *C. albicans*. This is also confirmed by the reduced kidney loads of mutant strains compared to the WT. However, it needs to be mentioned that further research is necessary to elucidate the role of morphogenesis and glycolytic flux in this hexokinase-dependent virulence during systemic infections. On the other hand, the reduction in virulence of strains with a deletion for *HXK1* (*hxk1*, *hxk1hxk2*, *hxk1 glk1 glk4*) is probably caused by the hyperfilamentation of this *hxk1* mutant (Singh et al., 2001). It is possible that due to its defect in switching morphology, the mutant cells are not as virulent as WT cells which is necessary for a normal virulence.

## Data Availability Statement

All datasets presented in this study are included in the article/supplementary material.

## Ethics Statement

The animal study was reviewed and approved by the KU Leuven Ethical Committee on animal experimentation: project number P023/2019.

## Author Contributions

PV, SW, KK, MR, and PP designed the experiments. SW, MR, and PP performed the experiments. SW and PV wrote the manuscript with additional input from KK, MR, and PP. All authors approved the final manuscript.

## Conflict of Interest

The authors declare that the research was conducted in the absence of any commercial or financial relationships that could be construed as a potential conflict of interest.
